# Sol*R*gene: an online database to explore disease resistance genes in tuber-bearing *Solanum *species

**DOI:** 10.1186/1471-2229-11-116

**Published:** 2011-08-18

**Authors:** Vivianne GAA Vleeshouwers, Richard Finkers, Dirk Budding, Marcel Visser, Mirjam MJ Jacobs, Ralph van Berloo, Mathieu Pel, Nicolas Champouret, Erin Bakker, Pavel Krenek, Hendrik Rietman, DirkJan Huigen, Roel Hoekstra, Aska Goverse, Ben Vosman, Evert Jacobsen, Richard GF Visser

**Affiliations:** 1 Wageningen UR Plant Breeding, Wageningen University and Research Centre, P.O. Box 386, 6700 AJ, Wageningen, The Netherlands; 2 Centre for BioSystems Genomics, P.O. Box 98, 6700 AB, Wageningen, The Netherlands; 3 Laboratory of Nematology, Wageningen University and Research Centre, Wageningen, The Netherlands; 4 Centre for Genetic Resources, Wageningen University and Research Centre, Wageningen, The Netherlands; 5 Centre of the Region Hana for Biotechnological and Agricultural Research, Department of Cell Biology, Faculty of Science, Palacky University, Slechtitelu 11, Olomouc, CZ-78371, Czech Republic

## Abstract

**Background:**

The cultivated potato (*Solanum tuberosum *L.) is an important food crop, but highly susceptible to many pathogens. The major threat to potato production is the Irish famine pathogen *Phytophthora infestans*, which causes the devastating late blight disease. Potato breeding makes use of germplasm from wild relatives (wild germplasm) to introduce resistances into cultivated potato. The *Solanum *section *Petota *comprises tuber-bearing species that are potential donors of new disease resistance genes. The aim of this study was to explore *Solanum *section *Petota *for resistance genes and generate a widely accessible resource that is useful for studying and implementing disease resistance in potato.

**Description:**

The Sol*R*gene database contains data on resistance to *P. infestans *and presence of *R *genes and *R *gene homologues in *Solanum *section *Petota*. We have explored *Solanum *section *Petota *for resistance to late blight in high throughput disease tests under various laboratory conditions and in field trials. From resistant wild germplasm, segregating populations were generated and assessed for the presence of resistance genes. All these data have been entered into the Sol*R*gene database. To facilitate genetic and resistance gene evolution studies, phylogenetic data of the entire Sol*R*gene collection are included, as well as a tool for generating phylogenetic trees of selected groups of germplasm. Data from resistance gene allele-mining studies are incorporated, which enables detection of *R *gene homologs in related germplasm. Using these resources, various resistance genes have been detected and some of these have been cloned, whereas others are in the cloning pipeline. All this information is stored in the online Sol*R*gene database, which allows users to query resistance data, sequences, passport data of the accessions, and phylogenic classifications.

**Conclusion:**

*Solanum *section *Petota *forms the basis of the Sol*R*gene database, which contains a collection of resistance data of an unprecedented size and precision. Complemented with *R *gene sequence data and phylogenetic tools, Sol*R*gene can be considered the primary resource for information on *R *genes from potato and wild tuber-bearing relatives.

## Background

Potato ranks third on the list of economically important food crops world-wide. However, potato is susceptible to many diseases and as a consequence, potato production depends on the application of enormous amounts of pesticides. The major disease in potato is late blight, which is caused by the oomycete pathogen *Phytophthora infestans *[1]. A durable control strategy based on natural resistance to late blight is of great importance.

Fortunately, ample genetic resistance is present in wild tuber-bearing *Solanum *species that belong to section *Petota*. The section *Petota *contains wild species that are distributed from the southwestern USA to central Argentina and adjacent Chile [2,3]. Potato breeders make use of this resource to introgress desired traits into cultivars [4-6]. Thus far, resistance (*R) *genes conferring resistance to *P. infestans *(*Rpi*) have been isolated from only a few wild *Solanum *species, i.e. *S. demissum, S. bulbocastanum *and *S. venturii *[7-13], and most of the resources in *Solanum *section *Petota *remain unexploited. While potato resistance breeding has so far been relatively unsuccessful, new approaches are emerging that use knowledge of effectors that are recognized by R proteins [14]. Late blight resistance as well as defeat of previously introgressed *R *genes is now better understood, and knowledge of effectors is being utilized in breeding and *R *gene deployment [15]. For consequent effector-based modern approaches, multiple *R *genes are required.

We have explored *Solanum *section *Petota *for *R *genes to *P. infestans*. Seeds from *Solanum *accessions were sown and individual genotypes are clonally maintained. This is in contrast to genebanks that maintain accessions as seeds. The rationale for our genotype-based studies is that many accessions are genetically highly dissimilar, since the majority of *Petota *species are self-incompatible out-breeders and heterozygous for many traits [2,16]. We tested the *Solanum *genotypes for resistance to a diverse set of well-characterized *P. infestans *strains. Indeed we found that in many cases variation of resistance occurs within accessions, and that resistant as well as susceptible genotypes occur, e.g. in *S. acaule *accession 425. Quantitative resistance data from routine disease tests using three different inoculation methods [17-19] were collected and stored in a database. Also pictures of the phenotypes observed in late blight field trials were included. This resulted in a unique data set of unprecedented size and precision.

For scientific as well as breeding purposes, it is important to have good insight in the taxonomy of relevant germplasm. However, in the *Solanum *section *Petota*, various taxonomic problems occur [2,3,20]. To resolve the phylogenetic relationships in the Sol*R*gene collection, all genotypes were subjected to a phylogenetic analysis based on AFLP [20,21]. A searchable interactive NJ tree is included in Sol*R*gene and permits identifying related germplasm for genetic studies and analyzing *R *genes evolution in comparison with species evolution.

*R *genes can be isolated using various strategies. Map-based cloning is a classic and thorough method to isolate *R *genes in potato which has proven successful for various *Rpi *genes, such as *R1, R2 *and *R3a *[7,8,22-24]. Allele-mining is a more high-throughput strategy to isolate genetic variants of *R *gene homologues, among which functional *R *genes can be detected. Strongly supported with rapid growing sequence information on *R *genes in the potato and tomato [25-32], efficient allele-mining for *R *gene homologues (*RGHs*) is dependent on availability of phenotyped genetic material, such as the Sol*R*gene collection. Recently, effector genomics is emerging as an efficient tool to accelerate *R *gene cloning, often in combination with small-scale genetic mapping and allele-mining [14].

Genetic studies are facilitated by generating populations. By making sexual crosses between resistant and susceptible *Solanum *genotypes, experimental (segregating) populations were produced. These are the basis for genetics studies that can lead to map-based cloning. For example, *S. venturii *was crossed with *S. neorossii *and the generated segregating population (7663) was used for the map-based cloning of *Rpi-vnt1.3 *[12]. Such cloned natural *R *genes are indicated as cisgenes if they originate from the potato plant itself or from crossable species. Due to the highly heterozygous and cross-pollinating nature of potato, genetic modification would be a major step in quickly achieving resistance by either using transgenesis or cisgenesis approaches. Cisgenesis is the combination of marker free transformation with only cisgenes [33].

The Sol*R*gene database was developed to provide a comprehensive dataset that can be used to explore *R *genes to potato pathogens in the *Solanum *section *Petota*. Major effort was attributed to *Rpi *genes acting against the late blight disease, but also other *R *genes were studied. A vast collection of disease phenotyping data, genetic data, allele-mining data of resistance genes against potato pathogens, and phylogenetic data complemented with an interactive tree tool are included, and are useful to unravel the genetic variation of *R *genes in the *Petota *gene pool. Hence, Sol*R*gene can be considered the primary resource for information on *R *genes of *Solanum *for the scientific community and potato breeders.

## Construction and content

### Data source of accessions

The current database version contains information on 1061 accessions (Table 1), obtained from different genebanks, i.e. the The Dutch-German Potato Collection at the Centre for Genetic Resources The Netherlands (CGN), The Commonwealth Potato Collection (CPC), The Groß Lüsewitz Potato Collection (GLKS), The potato Collection of the Vavilov Institute (VIR), The Potato Collection of the International Potato Center (CIP), and The US Potato Genebank (NRS). Accessions were originally collected from 14 countries in South and Central America, i.e. Argentina, Bolivia, Brazil, Chile, Colombia, Costa Rica, Ecuador, Guatemala, Mexico, Paraguay, Peru, USA, Uruguay and Venezuela, and geographical collection data are all included.

**Table 1:** Overview of the *So*l*R*genecollection with respect to resistance data, populations, and sequences obtained by allele-mining per phylogenetic group.

SolRgene Collection ^1^					LB resistance ^2^			Populations ^3^		Allele-mining ^4^			
**Subsection/Superseries**	**Series**	**Species**	**Accessions**	**Genotypes**	**vitro**	**leaf**	**field**	**total**	**Pi**	***Rpi-vnt1***	***Rpi-blb1***	***R2***	***Rx***

Estolonifera	Etuberosa	3	19	68	61	13	14	0	0	0	0	0	7
	Juglandifolia	4	9	40	20	27	7	0	0	0	0	0	0
Potatoe Stellata	Moreliformia	1	4	14	12	3	2	1	0	0	0	0	0
	Bulbocastana	6	37	182	165	66	54	44	6	0	0	0	6
	Pinnatisecta	9	44	264	223	119	96	75	5	0	0	0	0
	Polyadenia	2	10	66	51	31	16	20	2	0	0	0	0
	Commersoniana	5	21	60	20	3	4	4	0	0	0	0	0
	Circaeifolia	4	16	74	61	15	13	14	4	0	0	0	0
	Lignicaulia	1	3	6	1	0	0	0	0	0	0	0	0
	Yungasensa	8	62	360	284	116	93	83	6	4	0	0	18
	Megistacroloba	9	54	245	169	42	27	25	6	1	0	0	0
Potatoe Rotata	Cuneoalata	1	7	28	20	5	5	4	0	0	0	0	0
	Conicibaccata	23	73	286	186	107	27	14	2	0	0	0	0
	Piurana	10	22	81	50	26	11	9	2	0	0	0	0
	Demissa	10	39	188	173	76	68	22	24	0	0	13	14
	Maglia	1	4	14	14	1	1	0	0	0	0	0	0
	Tuberosa	101	527	2450	1949	586	426	417	95	38	12	0	1
	Tuberosa cult	2	19	112	111	11	10	16	1	3	0	0	1
	Acaulia	5	29	102	92	25	20	19	3	0	0	0	0
	Longipedicellata	6	45	298	273	80	79	136	29	0	8	5	0
	Not classified	5	17	71	1	15	13	129	3	3	0	3	0
		216	1061	5009	3936	1367	986	1032	188	49	20	21	47

^1 ^number of genotypes, accessions, species, subgroups belonging to the phylogenetic groups as classified by Hawkes [2]

^2 ^number of genotypes with resistance data obtained in various assays

^3 ^number of populations tested for segregation of resistance to late blight. Only the resistant parent was used to aggregate this classification

^4 ^number of RGA sequences in various allele-mining studies

The accessions represent *Solanum *section *Petota *and a few outgroup species. From these accessions, a set of 5009 genotypes was obtained from seeds, which are clonally maintained *in vitro *and are available upon request. The prevalence of 15 *R *genes or QTL is analyzed in the plant collection, with links to published papers.

### Phylogeny

Previously, we constructed a neighbor-joining (NJ) tree for 4929 genotypes [21]. Related to that dataset, we offer an interactive, searchable version of this NJ tree in Sol*R*gene. The different groups in the tree can be highlighted using the three letter species codes [34]. Neighbor-joining tree's, for a selected subsets of genotypes, can be calculated on-the-fly. The complete Sol*R*gene germplasm collection is also classified according to Hawkes [2], except for a few interspecific hybrids that were generated across series (Table 1).

### Crossability in tuber-bearing *Solanum *species

A total of 1032 successful crosses were made and information is stored within the Sol*R*gene database. The crosses were produced within and/or between species. In most cases, crosses within designated phylogenetic species groups were successful.

### Genotype-based resistance information

From each accession, on average five genotypes were characterized and the data of 5009 wild *Solanum *genotypes were stored independently in the database. Resistance data were generated using three different inoculation methods, i.e. high-throughput *in vitro *assays [17], detached leaf assays [18] and multi-year field trials [19] (Table 1). Pages explaining the disease assessment protocols supported with photographs are included. The majority (3936 genotypes) of the wild *Solanum *collection was tested with *P. infestans *isolate 90128 in the high throughput inoculation assay on *in vitro *plants. Part of the genotypes was tested in the laboratory using a routine detached leaf assay (1367 genotypes) with the *P. infestans *isolates 90128, IPO-C, or both. *Solanum *genotypes were also tested in field trials (986 genotypes) in 2005, 2007, or both, with *P. infestans *isolate IPO-C. A graphical representation of the resistance data facilitates a quick overview. From the field trails, 694 photographic images displaying symptoms on 362 genotypes are presented. Also two time lapse pictures of disease progress in the field are shown. Altogether, phenotyping of resistance resulted in 5 major sets of quantitative resistance data on the wild *Solanum *genotypes, and averages are presented too.

In addition to genotypes originating from genebank accessions, 7602 offspring genotypes from the generated populations were assessed for *P. infestans *resistance (see below). The offspring genotypes were generally tested in detached leaf tests, with four well-characterized *P. infestans *isolates, i.e. 90128, IPO-C, 88069, and H30P04, the reference isolate of the *P. infestans *genome sequence [35]. These resistance data provide information on segregation of specific *Rpi *genes.

### Map-based cloning using Sol*R*gene

To generate the required segregating populations for genetic mapping, resistant and susceptible plants were crossed. In total 1032 populations were generated. From these, 188 populations were phenotyped for resistance, and data were included in Sol*R*gene. Populations that are suitable for map-based cloning show a clear segregation between resistance and susceptibility in the F1, the so-called black & white segregation. Several of such segregating populations have entered into a pipe-line of genetic mapping and *R *gene isolation in our laboratory [12,14,32,36-38]. The first *R *genes to *P. infestans *(*Rpi*) from this resource have recently been cloned, such as *Rpi-vnt1.1, Rpi-vnt1.3 *[12] and *Rpi-sto1 *[14].

### Allele-mining in Sol*R*gene

Mining for late blight resistance genes *Rpi-vnt1, Rpi-blb1, Rpi-blb2, Rpi-blb3 *and its homolog *R2 *on the Sol*R*gene collection led to identification of an extensive number of RGH. Some of these were found functional and confer resistance to *P. infestans *[23,37-40]. A similar strategy was employed to identify four novel functional *Rx *genes (*Rx3*, *Rx4*, *Rx5 *and *Rx6*) from distinct *Solanum *species [41], which confer extreme resistance to PVX and share high sequence homology with *Rx1 *and *Rx2*.

### Database and web application

Sol*R*gene has been designed for simple and efficient data retrieval. It is composed of two major components: a relational database created using MySQL 5.1 and a web application which is implemented using PHP 5.2.6. The web interface runs on the Apache 2 Web server and is hosted on a Debian lenny linux server. The PHP scripts dynamically execute complex SQL queries to retrieve data from the database according to user criteria. HTML output, formatted using CSS style sheets, is generated to display results to the end-users. The relational MySQL database schema is segmented into seven main entities: Accession information, genotype information, population information, experiment information, disease observations, *R *genes and allele mining. Supporting tables were implemented containing e.g. information on origin of the accessions and availability of a genotype *in vitro*. Photos of many of the accessions are stored. Hyperlinks to Google Scholar and the NCBI gene bank records are provided for obtaining additional information on each accession/genotype. The Google Earth API is used to show the original collection site of the accession/group of selected accessions. All stored data is publicly accessible, so no authentication mechanism is built into the website.

## Utility and discussion

### Database web interface

**Figure 1:**
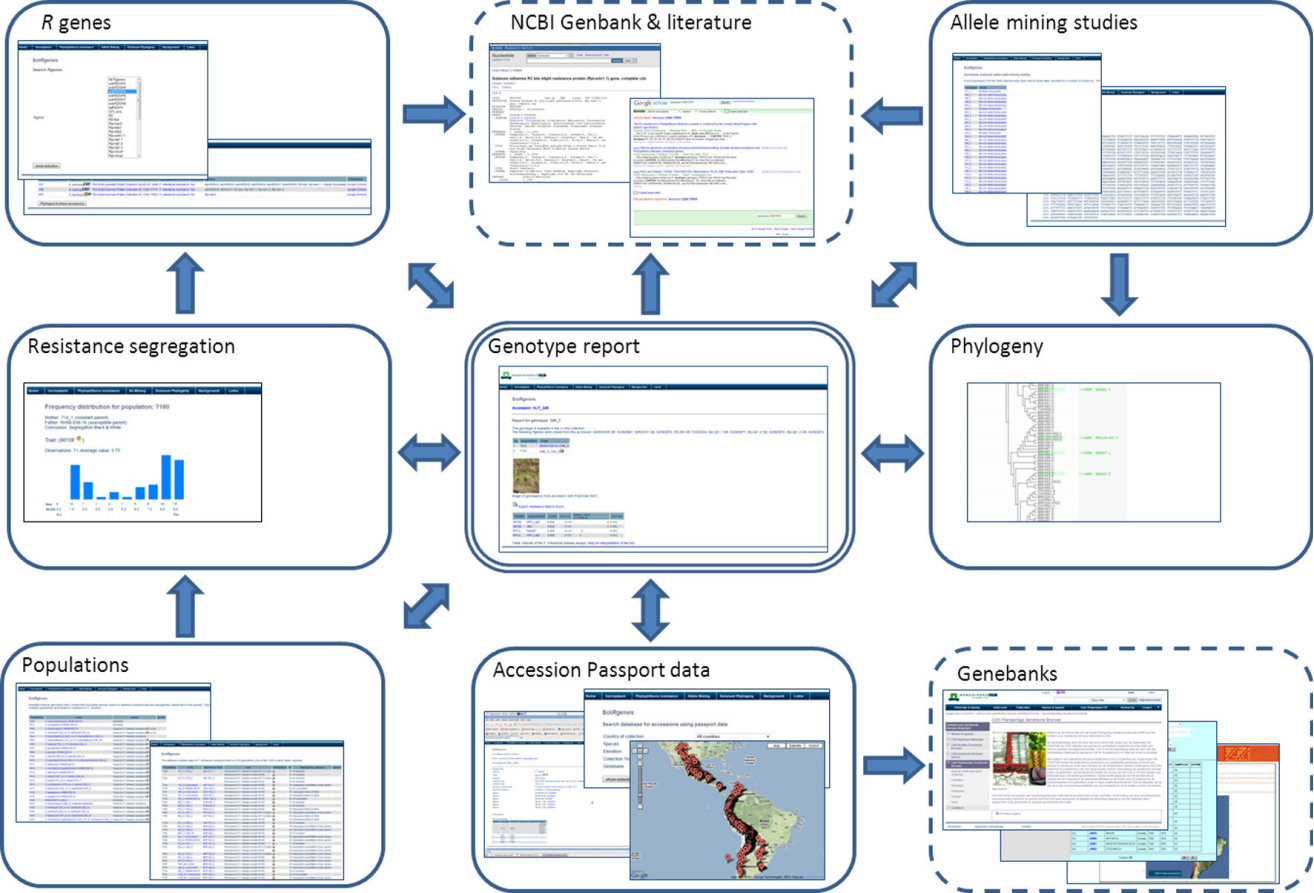
**Schematic representation of the data mining flow of the Sol*R*gene database**. The genotype is the central entity which links to all other types of data stored within the Sol*R*gene database (boxes with solid lines). Links to external resources are also provided (dashed boxes). The arrows between the different boxes show the directions in which the resource can be mined. The Sol*R*gene database can be searched using each entity as a starting point, and the resource can be mined consecutively in an iterative manner.

Sol*R*gene provides an interactive web-based graphical user-friendly interface to explore *Solanum *section *Petota *genotypes for resistance to *P. infestans *and late blight *R *genes. On every page, a menu tool bar appears, from which the germplasm can be searched for availability and overview data, resistance data, allele-mining data, and phylogeny. A menu for background information is also included (About). The genotype-based datasets of Sol*R*gene provide accurate data and allow direct phenotype - genotype associations that can be made from the various menu's (Figure 1).

The germplasm can directly be searched for genotypes, accessions, species, or phylogenetic classifications via the germplasm menu. Accessions can be searched, by passport data from different genebanks, or from visual geographic locations in Google Map (Figure 1). Outputs include lists with available germplasm and whether resistance data, populations are present, and whether *R *genes or RGH were amplified. An integrated hyperlink to Google Scholar enables quick searches for additional information on selected accessions on the world-wide web. Populations can be searched from the diverse available *Solanum *species in the germplasm menu. A phylogenetic tree, can be calculated on-the-fly, from selected genotypes.

The majority of the Sol*R*gene collection was phenotyped for resistance to *P. infestans*, and direct searches for resistance data can be performed via the *Phytophthora *resistance menu.

### How to get to *R *genes?

**Figure 2:**
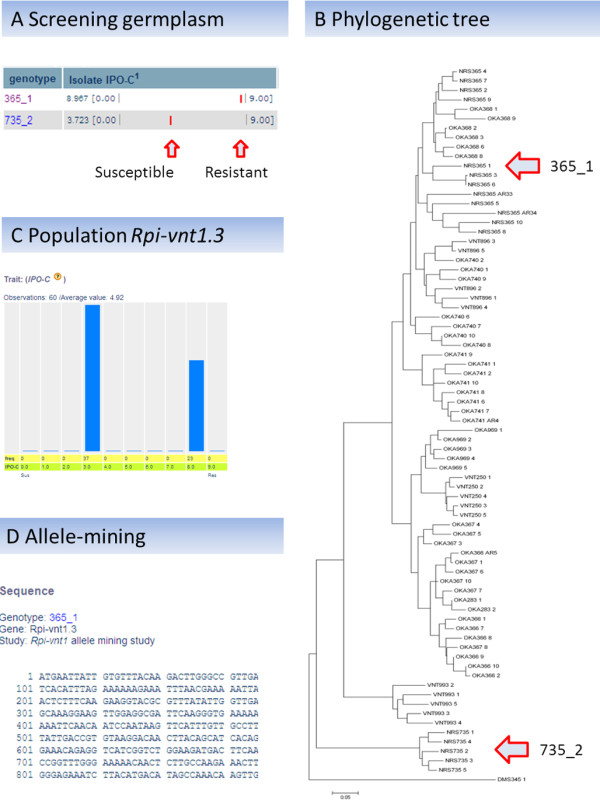
**Representation of cloning of *Rpi-vnt1.3 *using Sol*R*gene**. **A) **Resistant *Solanum *germplasm is selected based on screenings with *P. infestans *isolates. Results to isolate IPO-C are presented. Graphic representation facilitates quick overview of the quantitative resistance data: the red indicator shows the resistance level that ranges from fully susceptible (0, left) to fully resistant (9, right). **B**) Resistant genotypes (365_1) are crossed with related susceptible genotypes (735_2), selected from the phylogenetic tree. **C) **Population 7663 (365_1 × 735_2) is segregating for resistance to *P. infestans *isolate IPO-C, which is visualized by the frequency distribution for the offspring. The progeny part that contains *Rpi-vnt1.3 *is highly resistant (right bar, resistance level 8), whereas the progeny that lacks *Rpi-vnt1.3 *is moderately susceptible (left bar, resistance level 3). **D) **After genetic mapping, the *Rpi-vnt1.3 *was cloned, and used for allele-mining. In this menu, *R *genes and related sequences can be retrieved.

**Figure 3:**
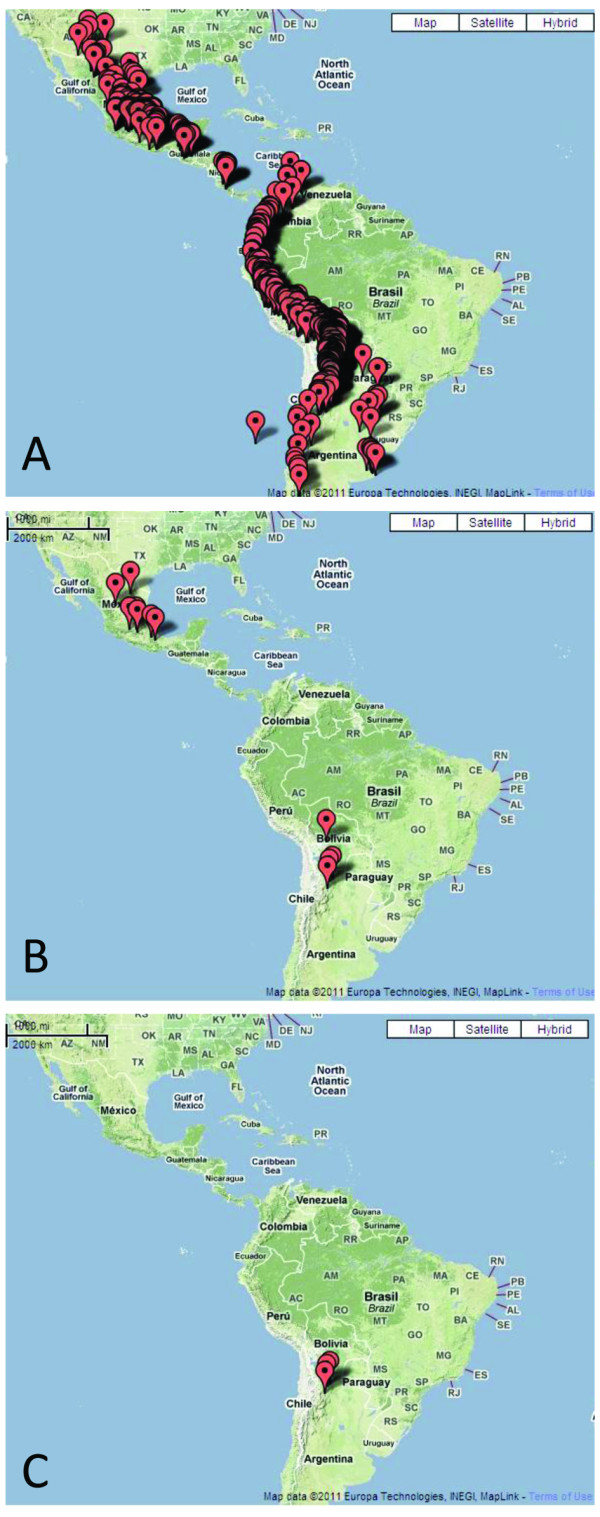
**Geographic origin of Sol*R*gene collection and identified late blight *R *genes**. **A**) The complete Sol*R*gene collection is originating from 14 countries in South, Central and North America. **B**) So-far identified *R *genes that confer resistance to *P. infestans *originate mainly from mountain regions in Central and South America. **C**) Accessions that contain *Rpi-vnt1 *homologs are restricted to Argentina.

After identifying resistant germplasm, genetics approaches are required to test whether the observed resistance can be attributed to *R *genes. For map-based cloning approaches, segregating populations can be selected and subjected to large-scale recombinant screenings. As an example, we present the cloning of *Rpi-vnt1.3 *[4] (Figure 2). First germplasm is screened for resistance to *P. infestans *isolates, and selected resistant genotypes are crossed with susceptible genotypes. These can be chosen using the phylogenetic tool. Obtained populations are tested for segregation of resistance to *P. infestans *isolates. Populations that clearly segregate for a specific *R *gene are used for map-based cloning purposes, sometimes in combination with allele-mining [4]. Allele-mining data for various *R *genes acting against *P. infestans *(*Rpi*), i.e. *R2*, *Rpi-vnt1*, *Rpi-blb1*, are included in Sol*R*gene and can be linked to relevant phenotyping data. In addition, allele-mining data for resistance genes against other *R *genes are included, i.e. *Rx *that confers resistance to potato virus X (PVX). Users can take advantage of all this information, and easily link their own plant material to Sol*R*gene, since *R *genes often originate from geographically restricted areas (Figure 3). Related germplasm may be identified using the Google Earth and passport data of genebanks. Also, related *R *genes are often identified in phylogenetically related material [40], and for this feature, the implemented phylogenetic tool can be applied.

### Future developments

The number of identified *R *genes to *P. infestans *and various other potato pathogens is increasing. In the near future, *R *genes and *R *gene allele-mining sequences will continue to be added (a.o. [32]), and thus, Sol*R*gene will provide an ever increasing source of *Solanum*-broad sequence information. Also, we welcome sequences or other contributions from the community to be added in this database. Sol*R*gene will also be linked to various databases including the full potato genome sequence in which our laboratory plays a leading role [25,27,42]. In addition, since the DNA sequence homology across *Solanum *species is high and ancestral *R *gene sequences are shared, also data for any other *Solanum *crops species like tomato, pepper and eggplant can be accommodated in the near future.

## Conclusions

So far, no survey involving such a large number of genotypes as well as phylogenetic coverage of tuber-bearing *Solanum *has been made assessable, uncovering numerous new resistance sources. Sol*R*gene is the first database that extends from phenotypic characterization to genetic dissection of the resistance by identification of functional *R *genes, and is regarded as the basis for potato *R *genes in the future. Sol*R*gene is easily searchable through a website interface and valuable for the scientific community as well as for applied breeding. The accurate genotypic data and the continued progress towards genetic analysis and *R *gene isolation distinguishes Sol*R*gene from gene bank databases. Essentially Sol*R*gene bridges the gap between well characterized plant material oriented databases and molecular sequence databases. In the near future, *R *genes, *R *gene allele-mining sequences, and AFLPs will continue to be added. Thus, the database will provide an ever increasing source of *Solanum*-broad sequence information, and linked to various databases including the full potato genome sequence.

## Availability and requirements

The Sol*R*gene database is freely accessible at http://www.plantbreeding.wur.nl/SolRgenes.

## List of abbreviations

*R*: resistance; *Rpi*: resistance to *P. infestans*; AFLP: Amplified Fragment Length Polymorphism.

## Competing interests

The authors declare that they have no competing interests.

## Authors' contributions

VGAAV designed the project, designed a relational database, integrated the data, and wrote the manuscript; RF worked on development and implementation of the web database, web layouts and contributed to writing the manuscript; DB carried out the majority of the technical work of disease testing in laboratory and field; MV carried out the vitro culturing *Solanum *plants and disease testing in vitro; MMJJ contributed to phylogenetic analysis based on AFLP; RvB for implementing the phylogenetic tools; MP, NC, PK and EB carried out the allele-mining of *Rpi-vnt1, R2, Rpi-blb1 *and *Rx*, respectively; HR contributed to field trials and generated the field photographs; DJH contributed to generating segregating populations; RH contributed *Solanum *accessions and information; AG contributed to *Rx *mining and writing the manuscript; BV contributed to phylogenetic analysis and writing the manuscript; EJ contributed to potato introgression breeding and provided valued discussions; RGFV conceived of the study, participated in its design and helped draft the manuscript. All authors have read and approved the manuscript.
